# Personality Traits and Physical Complaints in Patients With Acromegaly: A Cross Sectional Multi-Center Study With Analysis of Influencing Factors

**DOI:** 10.3389/fendo.2018.00391

**Published:** 2018-07-17

**Authors:** Anca Zimmermann, Rüdiger Zwerenz, Michael Droste, Christof Schöfl, Christian J. Strasburger, Ursula Plöckinger, Athanasia Ziagaki, Jürgen Honegger, Anne Dixius, Bledar Millaku, Gerrit Toenges, Manfred E. Beutel, Matthias M. Weber

**Affiliations:** ^1^Department of Endocrinology and Metabolic Diseases, 1. Medical Clinic, University Medical Center, Mainz, Germany; ^2^Clinic and Polyclinic for Psychosomatic Medicine and Psychotherapy, University Medical Center, Mainz, Germany; ^3^Endocrinology and Diabetology Praxis, Oldenburg, Germany; ^4^Center of Endocrinology and Metabolism, Bamberg, Germany; ^5^Clinical Endocrinology, Charité Universitätsmedizin Berlin, Berlin, Germany; ^6^Interdisciplinary Center of Metabolism: Endocrinology, Diabetes and Metabolism, Charité Universitätsmedizin Berlin, Berlin, Germany; ^7^Neurosurgery Clinic, University of Tübingen, Tübingen, Germany; ^8^Institute of Medical Biostatistics, Epidemiology and Informatics, University of Mainz, Mainz, Germany

**Keywords:** neuropsychological profile, physical complaints, acromegaly, disease activity, pituitary insufficiency

## Abstract

**Objective:** Acromegalic patients display a distinct neuropsychological profile and suffer from chronic physical complaints. We aimed to investigate in more detail these aspects in acromegalic patients, dependent on influencing factors like disease activity, age, sex, chronic medication, surgery, pituitary radiation, pituitary insufficiency and comorbidities.

**Design:** Cross sectional, multicentric.

**Methods:** 129 patients (M/W 65/64, 58.3 ± 12.7 years, 53/76 with active/controlled disease). Acromegalic patients completed the following inventories: NEO-FFI, IIP-D, and the Giessen Complaints List (GBB-24), after written informed consent. Age, sex, IGF-1 concentrations, comorbidities, treatment modalities and pituitary insufficiency were documented.

**Results:** Acromegalic patients or specific patient-subgroups were more agreeable, neurotic, exploitable/permissive, introverted/socially avoidant, non-assertive/insecure, nurturant and less open to experience, cold/denying, domineering, compared to normal values from the healthy population (controls). Multivariable analysis demonstrated that these overall results were due to the specific patient subgroups as patients on chronic medication, with arthrosis and pituitary insufficiency. Disease activity was only associated with the trait nurturant. Higher scores for introversion were associated with arthrosis. Lower domineering was independent of any disease- or treatment related variable or comorbidity. The GBB inventory showed overall higher scores in patients, with higher scores for exhaustion and general complaints being associated with pituitary insufficiency, coronary heart disease and history of malignancy in the multivariable analysis. Joint complaints were independent of any disease- or treatment- related variable.

**Conclusions:** We define new aspects of a distinct neuropsychological profile in patients with acromegaly, which are largely independent of disease activity. Chronic physical complaints are more pronounced in patients than in controls, with exhaustion and general complaints showing no association with disease activity.

## Introduction

Treatment goals in acromegaly are the achievement of hormonal control and remission of symptoms, removal of the adenoma with preservation of pituitary function, the cure of comorbidities, the prevention of recurrence and the improvement in quality of life (1–3).

Patients with acromegaly have been reported to display specific personality traits and physical complaints. Sievers et al. compared patients with acromegaly with patients with pituitary adenomas and healthy controls. They reported that patients with acromegaly were more avoidant, neurotic and socially conformant, and suffered from anticipatory worries, pessimism and fear of uncertainty, fatigability and asthenia, compared to healthy controls. These traits were also encountered in patients with pituitary adenomas. However, a reduced impulsiveness and novelty seeking behavior was specific for acromegalic patients and was independent of age, sex, adenoma type, treatment modality, pituitary insufficiency and hormonal control (4).

Neuropsychological alterations can negatively impact on other domains like quality of life, cognitive function and subjective well-being. Interestingly, many chronic alterations in patients with acromegaly persist despite hormonal control. Previous studies reported a reduced quality of life even if biochemical control has been reached (5–8). This might be due to joint-related comorbidities (9) or previous radiotherapy, which in turn can lead to social isolation, reduced motivation, depression and anxiety (10, 11). Recent data showed that reduction in quality of life is associated with depression and anxiety rather than with biochemical control or other possible predictors like age, sex, tumor size, disease duration, treatment type and comorbidities (5).

Pereira et al. observed that, compared to subjects with other pituitary tumors, schwannomas or chronic pain, patients with acromegaly displayed more cognitive impairment and personality disorders. In addition, one third of these patients were diagnosed with anxiety and depression. These findings were common for patients with active and controlled disease with long-lasting remission, and possibly caused by irreversible alterations of the central nervous system due to long term growth hormone (GH) excess or by residual disabilities like chronic arthrosis-associated pain (12, 13).

Besides these alterations, well-being in acromegalic patients may be negatively affected by a variety of complaints. Patients with acromegaly revealed worse scores on physical functioning, reduced activity, vitality, and general health perception, if they needed postoperative treatment with long-acting somatostatin analogs (6). Furthermore, joint problems were not related to GH and IGF-1 levels, active disease duration, or age (9). Long-term cured acromegaly patients had high pain scores of the spine, knee and hip which limited physical functioning (14).

Based on the current literature, it is not clear which factors influence these alterations of personality, mood and physical wellbeing. Therefore, we aimed to analyze in a comprehensive multi-center non-interventional study personality, relationship patterns and physical complaints in 129 patients with acromegaly, compared to standardized manual data and the impact of influencing factors like age, sex, disease activity, pituitary insufficiency, disease duration, treatment variables and comorbidities on these parameters.

## Patients and methods

### Study design

This is a cross-sectional multicenter non-interventional study, conducted at the Department of Endocrine and Metabolic Diseases, 1st Medical Clinic and Polyclinic, University of Mainz, Germany. Patients were recruited from six German endocrine centers. Inclusion criteria were: age >18 years, diagnosis of acromegaly, willingness to participate in the study, written informed consent. Exclusion criteria were: psychiatric disorder; psychiatric medication (antidepressants, antipsychotics, sedatives); excessive alcohol consumption (>30 g/day in men and >20 g/day in women) or drug addiction; pregnancy or lactation; a language barrier.

### Patients

The patients were seen either in the outpatient Dept. of Endocrinology and Metabolic Diseases Mainz or in one of the other five cooperating centers for a standardized clinical assessment with physical examination and laboratory testing. The patients were asked at a regular follow-up visit to complete three questionnaires. Additional information was documented by the physician regarding age, sex, acromegaly status with definition of disease activity: hormonally uncontrolled (before or under treatment); and hormonally controlled (under or after treatment). Hormonal control was defined according to consensus guideline recommendations as GH levels < 1 μg/l during a glucose tolerance test over 2 h and IGF-1 levels in the age- and sex-matched normal range (2) and was applied as such in all participating centers. Treatment was defined as surgery, chronic medication against GH-excess or pituitary radiation. Further data were: age at diagnosis; secretion pattern (GH excess only or mixed GH and prolactin overproduction); current IGF-1 value (ng/ml), current or previous treatment of GH excess with duration (surgery, medication, radiotherapy), comorbidities (coronary heart disease, arterial hypertension, diabetes mellitus, cancer history, arthrosis); current medication and dosage, including hormonal treatment for coexisting pituitary deficiency. The data were documented in an anonymized form.

The project was approved by the Medical Ethics Committee of the University of Mainz. All patients provided written informed consent. We included 129 patients, fulfilling the above criteria.

### Hormonal measurements

To avoid methodological and assay differences among the participating centers, a single additional serum sample was collected from each patient during routine laboratory testing. This was frozen at −20°C and sent to the University of Mainz for standardized measurement of IGF-1 in the Institute for Clinical Chemistry and Laboratory Medicine. In our center, IGF1 measurement of all samples has been performed with the IGF1 kit from Siemens on a Siemens Immulite 2000 device, with a chemiluminescence immunoassay method.

### Psychological assessment

NEO-FFI is a psychological personality inventory developed by Costa and McCrea, containing in its original form five subscales investigating neuroticism, extraversion, openness to experience, agreeableness and conscientiousness. The inventory contains 12 items/subscales and a total of 60 items, scored between 0 and 4. The results were interpreted and compared to the general population based on the manual's data (15).

IIP-D is a self-reported instrument that identifies interpersonal problems. We used the short form of the German version with 64 items, based on a circumplex model evaluating control/dominance- and fondness. The following characteristics were analyzed: domineering; vindictive/competing; cold/denying; introverted/socially avoidant; non-assertive/insecure; exploitable/permissive; nurturant (i.e., relating to the fact of taking care of or the ability to do so, in both a physical and emotional manner); intrusive (i.e., a typically unwelcome behavior, interrupting and disturbing to others) (16). The score for each item ranged between 0 and 4. The sum of the scale value was divided by the number of items/subscale to obtain the normed individual subscale value. For comparison with the general population we used standardized data from the handbook, based on a representative German sample of individuals aged between 14 and 98 years. The overall individual IIP-value was obtained by the sum of all eight scales, divided by eight.

### Assessment of physical complaints

The Giessen Subjective Complaints List (GBB-24) quantifies physical complaints grouped in four subscales: exhaustion (E), gastric (G), joint (J), and heart (H) complaints. Each subscale comprises six questions which are ranked from 0 to 4. The highest subscale value and herewith the highest degree of the specific complaint can be 24. The sum of all subscales yields the general complaints score (total GBB) and can reach from 0 to 96. The inventory was standardized using a sample of 2,182 subjects aged 18–60 years, representative for the German population. The scale norms were also subdivided according to sex and age (17).

### Controls

The inventory results of the patients were compared to normative values of the general population, which are provided by the respective inventory-manual in the form of age- and sex-specific means and standard deviations for each subscale (controls).

### Statistical analysis

Statistical analyses were subdivided into three parts: First, inventory results were compared to manual-based normative values of the general population (controls; see above). In each subgroup being defined by age and sex, the patient's score was compared to the respective mean for the general population by performing a one sample t-test (for normally distributed data) or a sign test (for not normally distributed data).

Second, we examined differences between the general population (normal manual data for the healthy population; controls) and several patient-subgroups that were prescribed by binary disease- or comorbidity-related variables (such as active vs. controlled disease, surgery yes/no, radiation yes/no, etc.). To adjust for age and sex as confounders within these comparisons, the patients' subscale-values were transformed to Z-scores by subtracting the mean and dividing by the standard deviation of the respective normative age-/gender-subgroup. For comparisons between patient-subgroups (being defined by disease- or comorbidity-related variables) and the general population, we analyzed if the patients' Z-scores deviate from zero by using a one-sample Wilcoxon signed rank test.

Third, we compared the patients among each other by using multivariable linear regression analysis (MV). For each questionnaire we used the main subscale-scores as well as the total score (where available) as the respective dependent variable. As explanatory variables, we considered those that are linked to the disease/disease activity (active vs. controlled status, medication, surgery, radiation, pituitary insufficiency) and variables to control for confounding due to comorbidities - such as coronary heart disease, arterial hypertension, diabetes, history of malignancy and arthrosis. Regression techniques were used to assess if modeling assumptions were fulfilled. For each multivariable regression model, we present the coefficients along with 95% confidence intervals and the *p*-values of a two-sided test.

Statistical analyses were performed with the R environment for statistical computing and graphics, version 3.4.4 (R Core Team, 2017). Our complete data analysis is exploratory. For all tests we used a 0.05 level to define subgroup-differences as statistically noticeable and relevant. However, due to the large number of tests, *p*-values should be interpreted with caution and in connection with effect estimates.

## Results

The patients' characteristics of the 129 included patients are presented in Table 1. The distribution in the four groups, according to the hormonal activity and treatment status at the time of evaluation was: uncontrolled before any treatment (*n* = 17, 13%, IGF-1 = 719.8 ± 281.9 ng/ml); uncontrolled under treatment (chronic medication against GH-excess; *n* = 36, 28%, IGF-1 = 417.8 ± 224.9 ng/ml); controlled under treatment (chronic medication against GH-excess; *n* = 27, 21%, IGF-1 = 177.6 ± 73.1 ng/ml); controlled after treatment (surgery ± radiation, chronic medication; *n* = 49, 38%, IGF-1 = 175.9 ± 66.1 ng/ml). Some patients had more than one comorbidity. The most common comorbidity was arthrosis (53.5%), followed by arterial hypertension (36.4%), diabetes mellitus (15.5%), coronary heart disease (12.4%), and a history of malignancy (10%).

**Table 1:** Patients characteristics.

**Parameters**	**Distribution**
Age (years)	58.3 ± 12.7 (30–86)
Time since diagnosis (years)	13.4 ± 8.1 (0–23)
M/F	65/64
GH/GH and prolactin	120/9
Active disease/biochemical control	53 (41.1%)/76 (58.9%)
Complete/partial pituitary insufficiency	10 (7.8%)/37 (28.7%) thyreotrope insufficiency (*n* = 27), corticotrope insufficiency (*n* = 20), gonadotrope insufficiency (*n* = 23)
Treatment	112 (yes)/17 (no)
Pituitary surgery	104/112; 92.9%; > 1 surgery (*n* = 17/112; 15.2%)
Medication	68/112 (60.7%)
	Somatostatin analogs: 36 (32.1%)
	Dopamin agonists: 7 (6.3%)
	Pegvisomant: 2 (1.8%)
	Secvential or combined: 23 (20.5%)
Radiotherapy	16/112 (14.3%)
Comorbidities	Arthrosis: 69/129 (53.5%) Arterial hypertension: 47/129 (36.4%) Diabetes mellitus: 20/129 (15.5%) Coronary heart disease: 16/129 (12.4%) History of malignancy: 13/129 (10.0%)

*Continuous data are presented as mean ± 1 standard deviation (range), categorical data are presented as counts/ number of subjects (%)*.

### Neuropsychological profile, according to the NEO-FFI inventory

The relevant age- and sex-specific differences between all patients with acromegaly, irrespective of hormonal activity, and controls are given for the NEO-FFI inventory in Table 2. Male patients aged 30–49 years had higher scores of agreeableness (*p* = 0.005), while male patients older than 50 years and female patients older than 30 years showed a lower openness to experience *p* = 0.024 and *p* < 0.001, respectively).

**Table 2:** Age- and sex-specific differences between patients and controls based on the NEO-FFI inventory.

**Age (years)**	**Parameter**	**Males**			**Females**		
		**Patients**	**Controls**	***p***	**Patients**	**Controls**	***p***
30–49	Neuroticism	21.10 ± 9.3	18.8 ± 7.8	0.274	19.76 ± 7.4	22.04 ± 8.28	0.131
n males = 25 patients /1035 controls)	Extraversion	25.52 ± 8.1	26.82 ± 6.66	0.471	28.93 ± 4.9	27.87 ± 6.41	0.280
*n* females = 28 patients/1992 controls)	**Openness to experience**	30.19 ± 5.0	30.68 ± 6.56	0.712	26.81 ± 4.9	31.34 ± 6.15	*<**0.001***
	**Agreeableness**	31.81 ± 4.0	29.02 ± 5.1	***0.005***	32.61 ± 5.7	31.1 ± 5.2	0.182
	Conscientiousness	31.48 ± 7.3	31.41 ± 6.86	0.967	34.0 ± 6.0	32.43 ± 6.54	0.196
≥50	Neuroticism	19.19 ± 6.6	19.5 ± 7.15	0.893	20.98 ± 5.4	21.83 ± 7.7	0.376
n males = 42 patients/403 controls	Extraversion	24.01 ± 5.7	25.43 ± 6.15	0.129	26.21 ± 4.9	27.1 ± 6.15	0.308
*n* females = 34 patients/713 controls	**Openness to experience**	26.47 ± 6.2	28.84 ± 6.12	***0.024***	27.03 ± 4.3	29.97 ± 6, 1	*<**0.001***
	Agreeableness	30.71 ± 4.9	29.54 ± 4.97	0.154	32.82 ± 5.5	32.1 ± 4.8	0.432
	Conscientiousness	32.39 ± 5.6	33.37 ± 5.97	0.291	34.64 ± 5.5	33.1 ± 5.6	0.118

*Continuous data are presented as mean ± 1 standard deviation. P < 0.05 defined subgroup-differences as statistically noticeable and relevant*.

To gain more insight into the differences between patients and controls, we performed an age- and sex- independent analysis with Z-score calculations, considering the following parameters: disease activity, surgery, pituitary radiotherapy, medication for disease control, pituitary insufficiency, comorbidities (coronary heart disease, arterial hypertension, diabetes mellitus, history of malignancy, arthrosis). Differences among patients, considering the abovementioned parameters, including also age and sex, were explored by multivariable analysis (MV).

**Figure 1:**
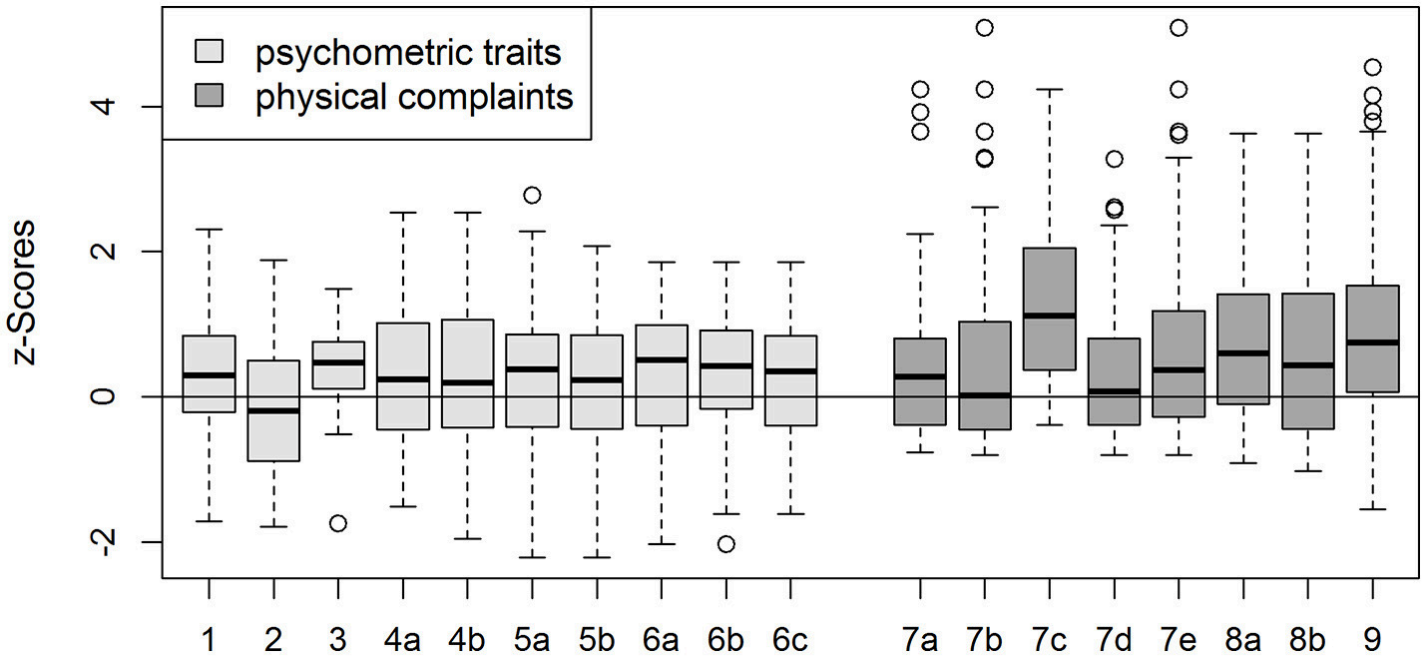
Z scores (mean/median) for different neuropsychological traits and physical complaints, which differed from controls in an age- and gender- independent comparison, with respect to disease- and treatment associated factors as well as comorbidities. Z-score formation implies that values of age- and sex-matched controls would be 0 in mean/median. 1—*agreeable* in patients on chronic medication (*Z* = 0.31 ± 0.90); 2—*cold/denying* in patients with pituitary insufficiency (*Z* = −0.24 ± 0.83); 3—*introvert/socially avoidant* in patients who received pituitary radiation (*Z* = 0.34 ± 0.74); 4—*non-assertive/insecure* in patients (a) on chronic medication (*Z* = 0.27 ± 0.93), (b) with arthrosis (*Z* = 0.25 ± 0.96); 5—*exploitable/permissive* in patients (a) on chronic medication (*Z* = 0.28 ± 0.99), (b) with arthrosis (*Z* score = 0.20 ± 0.88); 6—*nurturant* in patients with (a) active disease (*Z* = 0.28 ± 0.94), (b) on chronic medication (*Z* = 0.28 ± 0.9), (c) with arthrosis (*Z* = 0.26 ± 0.85); 7—*gastric complaints* in patients with (a) active disease (*Z* = 0.51 ± 1.33), (b) on chronic medication (Z = 0.51 ± 1.33), (c) with history of malignancy (*Z* = 1.33 ± 1.28), (d) with arthrosis (*Z* = 0.34 ± 0.95), (e) with pituitary insufficiency (*Z* = 0.77 ± 1.47); 8—*heart complaints* in patients (a) with active disease (*Z* = 0.77 ± 1.15), (b) on chronic medication (*Z* = 0.58 ± 1.21); 9, *total GBB* in patients with comorbidities overall (*Z* = 0.89 ± 1.27).

A subgroup comparison, according to the specified variables showed that patients on chronic medication had higher scores of agreeableness than controls, independent of age and sex (*p* = 0.008; Figure 1: 1). No relevant differences were seen between patients not meeting these criteria and controls.

Among patients, the MV of the NEO-FFI inventory showed lower openness to experience in those with history of malignancy (*p* = 0.047) (Supplementary Table 1).

A further subgroup analysis showed higher neuroticism scores in hormonally controlled patients having at least one pituitary insufficiency compared to those without pituitary insufficiency (22.0 ± 6.4 vs. 18.7 ± 7.4, *p* = 0.042). Neuroticism was also higher in hormonally controlled female patients with at least one pituitary insufficiency, compared to those without pituitary insufficiency (22.9 ± 7.0 vs. 18.1 ± 7.2, *p* = 0.044) and between female patients with controlled disease on specific medication (somatostatin analogs/dopamine agonists/pegvisomant) and those with controlled disease without chronic medication (21.2 ± 7.2 vs. 17.8 ± 7.1, *p* = 0.054). Furthermore, female patients with at least one pituitary insufficiency had lower values for agreeableness (29.9 ± 5.6 vs. 35.0 ± 4.6, *p* = 0.007) compared to female patients without pituitary insufficiency (data not shown). Since dopamine agonists can cause neuro-psychological adverse effects themselves, we subdivided patients on chronic medication in those who received dopamine agonists (*n* = 7, all in combination with SSA) and those who did not receive dopamine agonists in their treatment regimen (*n* = 61). Neuroticism scores were 21.6 ± 6.6 in the dopamine-subgroup and 21.1 ± 6.2 in the patients not receiving dopamine agonists (*p* = 0.884).

Taken together, the NEO-FFI showed that younger male patients had higher scores of agreeableness while older male patients and younger female patients demonstrated a lower openness to experience.

### Neuropsychological profile, according to the IIP-D inventory

Table 3 shows the relevant differences of parameters of the IIP-D-inventory between patients and controls, according to age and sex. Male patients aged 35–44 years were more exploitable/permissive than controls (*p* = 0.011), while female patients of this age group were more introverted/socially avoidant (*p* < 0.001). Male and female patients aged 45–54 years were less domineering than controls (*p* = 0.031 for men and *p* = 0.017 for women). Of note, there were no obvious differences in this inventory between patients and controls aged 55–64 years, nor in those older than 65 years (13 female patients, no male patients, 219 controls; data not shown).

**Table 3:** Age- and sex-specific differences between patients and controls based on the IIP-D inventory.

**Age (years)**	**Parameter**	**Males**			**Females**		
		**Patients**	**Controls**	***p***	**Patients**	**Controls**	***P***
35–44	Domineering	0.99 ± 0.5	1.12 ± 0.66	0.335	1.24 ± 0.7	1.04 ± 0.65	0.341
*n* males = 17 patients /255 controls	Vindictive/competing	0.19 ± 0.4	1.13 ± 0.63	0.560	0.96 ± 0.7	1.01 ± 0.55	0.811
*n* females = 17 patients/316 controls	Cold/denying	1.27 ± 0.6	1.21 ± 0.64	0.719	1.14 ± 0.68	0.96 ± 0.5	0.117
	**Introverted/socially avoidant**	1.59 ± 0.8	1.23 ± 0.74	0.112	1.27 ± 0.76	0.82 ± 0.3	*<**0.001***
	Non-assertive/insecure	1.76 ± 1.0	1.32 ± 0.76	0.121	1.23 ± 0.5	1.46 ± 0.78	0.791
	**exploitable/permissive**	1.78 ± 0.6	1.34 ± 0.68	***0.011***	1.56 ± 0.5	1.49 ± 0.68	0.612
	Nurturant	1.73 ± 0.6	1.47 ± 0.66	0.137	1.60 ± 0.5	1.57 ± 0.65	0.285
	Intrusive	1.22 ± 0.6	1.19 ± 0.65	0.849	1.42 ± 0.7	1.20 ± 0.62	0.285
45–54	**Domineering**	0.86 ± 0.6	1.17 ± 0.67	***0.031***	0.63 ± 0,5	1.01 ± 0.62	***0.017***
*n* males = 23 patients/196 controls	Vindictive/competing	0.99 ± 0.4	1.10 ± 0.58	0.815	0.80 ± 0.4	1.01 ± 0.61	0.128
*n* females = 17 patients/222 controls	Cold/denying	1.16 ± 0.6	1.24 ± 0.71	0.609	0.93 ± 0.5	1.10 ± 0.67	0.231
	Introverted/socially avoidant	1.35 ± 0.8	1.27 ± 0.75	0.662	1.16 ± 0.8	1.24 ± 0.72	0.710
	Non-assertive/insecure	1.41 ± 0.7	1.35 ± 0.78	0.694	1.66 ± 0.6	1.48 ± 0.79	0.354
	Exploitable/permissive	1.43 ± 0.5	1.36 ± 0.61	0.568	1.60 ± 0.5	1.43 ± 0.66	0.265
	Nurturant	1.45 ± 0.4	1.50 ± 0.57	0.589	1.52 ± 0.5	1.53 ± 0.58	0.952
	Intrusive	1.00 ± 0.5	1.16 ± 0.60	0.195	0.92 ± 0.5	1.20 ± 0.58	0.064
55–64	Domineering	1.03 ± 0.9	1.10 ± 0.65	0.759	0.95 ± 1.0	1.03 ± 0.64	0.581
*n* males = 18 patients /232 controls	Vindictive/competing	1.40 ± 0.7	1.10 ± 0.59	0.127	0.92 ± 0.7	0.98 ± 0.60	0.879
*n* females = 17 patients /293 controls	Cold/denying	1.46 ± 0.8	1.26 ± 0.68	0.382	0.98 ± 0.8	1.22 ± 0.68	0.092
	Introverted/socially avoidant	1.47 ± 0.8	1.24 ± 0.68	0.277	1.34 ± 0.6	1.31 ± 0.75	0.870
	Non-assertive/insecure	1.57 ± 0.8	1.43 ± 0.73	0.530	1.61 ± 0.9	1.50 ± 0.74	0.647
	Exploitable/permissive	1.58 ± 0.7	1.44 ± 0.65	0.057	1.66 ± 0.8	1.55 ± 0.68	0.631
	Nurturant	1.64 ± 0.5	1.52 ± 0.58	0.180	1.77 ± 1.0	1.62 ± 0.61	0.598
	Intrusive	1.13 ± 0.7	1.19 ± 0.58	0.771	1.15 ± 0.9	1.22 ± 0.60	0.798

*Continuous data are presented as mean ± 1 standard deviation. P < 0.05 defined subgroup-differences as statistically noticeable and relevant*.

A subgroup comparison, according to the mentioned variables, revealed that patients with pituitary insufficiency were less cold/denying (*p* = 0.034), patients who received pituitary radiation were more introverted/socially avoidant (*p* = 0.027) and patients on chronic medication or suffering from arthrosis were more non-assertive/insecure than controls (*p* = 0.029 and 0.044, respectively), independent of age and sex. Furthermore, scores for exploitability/permissiveness were higher in patients on chronic medication (*p* = 0.022) or in those suffering from arthrosis (*p* = 0.047), compared with controls. The trait *nurturant* was more pronounced in patients with active disease (*p* = 0.021), on chronic medication (*p* = 0.009) and in those suffering from arthrosis (*p* = 0.009), in the age- and sex-independent comparison with controls (Z scores in Figure 1: 2–6). No differences were seen between patients not meeting these criteria and controls.

Among the patients, those suffering from arthrosis were more introverted/socially avoidant (*p* = 0.022), as shown by MV (Supplementary Table 2).

Taken together, the IIP-D Inventory demonstrated age specific differences between AP and controls. The younger female patients showed more introvertion/social avoidance, while younger male patients were more exploitable/permissive. The middle aged group was significantly less domineering.

### Physical complaints, according to the GBB inventory

The GBB inventory has been completed only by 124/129 patients. In patients younger than 40 years, exhaustion was higher in patients than controls for both sexes, while joint and heart complaints were more often described in female patients. With increasing age exhaustion, joint and heart complaints were higher in patients. Finally, in those over 60 years of age gastric and joint complaints were increased in patients compared to controls, while exhaustion was higher only in male patients. Heart complaints at this age were comparable to controls. Total GBB scores were overall higher in patients, except male patients younger than 40 years (Table 4).

**Table 4:** Age- and sex-specific differences between patients and controls based on the GBB inventory.

**Age (years)**	**Parameter**	**Males**			**Females**		
		**Patients**	**Controls**	***P***	**Patients**	**Controls**	***P***
≤ 40 years	Exhaustion	9.71 ± 7.04	2.52 ± 3.8	**0.035**	8.75 ± 5.84	3.32 ± 3.8	**0.034**
*n* males = 9 patients/255 controls	Gastric complaints	1.28 ± 1.11	1.52 ± 2.6	0.59	6.0 ± 5.34	1.76 ± 2.8	0.060
*n* females = 11 patients/316 controls	Joint complaints	5.82 ± 3.48	3.31 ± 3.8	0.101	8.87 ± 3.68	4.0 ± 3.9	**0.007**
	Heart complaints	1.0 ± 1.52	1.19 ± 2.7	0.75	4.25 ± 3.45	1.3 ± 2.4	**0.046**
	Total score	17.85 ± 10.73	8.53 ± 11.11	0.061	27.8 ± 15.9	10.38 ± 11.1	**0.017**
40–59 years	Exhaustion	7.86 ± 4.61	3.18 ± 3.7	<**0.001**	10.13 ± 7.20	4.21 ± 4.5	<**0.001**
*n* males = 20 patients/196 controls	Gastric complaints	2.91 ± 2.60	2.26 ± 3.0	0.24	3.03 ± 3.5	2.16 ± 3.0	0.118
*n* females = 35 patients/222 controls	Joint complaints	8.82 ± 2.60	4.89 ± 4.1	**0.003**	9.83 ± 6.39	6.35 ± 4.6	**0.006**
	Heart complaints	3.65 ± 3.15	1.88 ± 3.0	**0.013**	5.13 ± 4.46	2.27 ± 3.3	**0.002**
	Total score	23.26 ± 13.57	12.2 ± 11.8	**0.001**	27.93 ± 18.65	14.9 ± 13.0	**0.001**
>60 years	Exhaustion	9.0 ± 5.11	4.69 ± 4.2	**0.001**	7.77 ± 5.45	6.17 ± 4.4	0.091
*n* males = 21 patients/232 controls	Gastric complaints	4.08 ± 2.59	2.74 ± 3.4	**0.021**	4.28 ± 4.69	2.44 ± 3.2	**0.026**
*n* females = 19 patients/293 controls	Joint complaints	9.39 ± 4.94	6.89 ± 4.1	**0.024**	10.57 ± 5.59	8.48 ± 4.7	**0.034**
	Heart complaints	4.21 ± 3.24	3.39 ± 3.7	0.235	4.51 ± 4.05	3.78 ± 3.7	0.281
	Total score	26.69 ± 12.10	17.71 ± 13.1	**0.002**	26.85 ± 17.16	20.70 ± 13.3	**0.041**

*Continuous data are presented as mean ± 1 standard deviation. P < 0.05 defined subgroup-differences as statistically noticeable and relevant*.

In the age- and sex- independent comparison between patients and controls, according to disease-specific factors and comorbidities, we noticed that gastric complaints were more pronounced in patients with active disease (*p* = 0.023), on chronic medication (*p* = 0.045), with a history of malignancy (*p* = 0.005), with arthrosis (*p* = 0.032) and with pituitary insufficiency (*p* = 0.003), compared with controls. Heart complaints in patients exceeded those found in controls, when the disease was active (*p* < 0.001) or chronic medication was present (*p* = 0.002). No relevant differences were seen between patients not meeting these criteria and controls. The total GBB-value was higher in patients with comorbidities overall (p < 0.001) (Figure 1: 7–9).

MV revealed that among patients, exhaustion was more pronounced in those with pituitary insufficiency (*p* = 0.015) and coronary heart disease (*p* = 0.009) (Supplementary Table 3). A history of malignancy was associated with more heart complaints (*p* = 0.006). Higher total GBB scores were registered in patients with pituitary insufficiency (*p* = 0.043), and a history of malignancy (*p* = 0.034). Interestingly, a history of pituitary surgery or sellar radiotherapy was not associated with more physical complaints among patients, nor was disease activity associated with exhaustion, joint complaints or the total GBB score.

Taken together the GBB inventory demonstrated less sex differences than seen in the previous tests. Complaints increased by age with exhaustion being the leading complaint throughout, while gastric, joint and heart complains increased age-related. Beyond 40 years of age the total GBB score was higher at each age group compared to normal values.

## Discussion

Our multicenter study in acromegalic patients found specific personality characteristics and relationship patters. Overall, patients suffered from a high somatic symptom load.

### Neuropsychological profile, according to the NEO-FFI and the IIP-D inventories

The analysis of the neuropsychologic profile confirmed some already described traits and identified new elements. Patients or specific subgroups were less open to experience, more agreeable, permissive and introvert and less domineering, compared to manual normal values for the healthy population. Lower openness to experience was associated with a history of malignancy. Lower dominancy was not associated with any disease variable, treatment modality or comorbidity, suggesting possibly an intrinsic disease-associated personality alteration. Only introversion associated with one comorbidity in MV (arthrosis).

Our large acromegalic cohort displayed significantly lower scores for openness to experience in the NEO-FFI inventory and thus confirmed data from Sievers et al. who conducted a cross sectional study on 70 acromegalic patients. Their patients had a psychological profile that could be similarly observed in patients with non-functioning pituitary adenomas. However, patients with GH-producing adenomas showed specific traits like lower impulsiveness and reduced novelty seeking behavior (4). Previous data pointed to increased rates of affective disorders in acromegaly. Affective disorders were found in 34.6% of the patients with acromegaly, compared to 21% of patients with and 11% of those without chronic somatic disorders. Rates were even higher in those acromegalic patients who needed additional treatment after surgery (18). The same authors showed that depression and anxiety negatively impacted quality of life in acromegaly (5).

Neuroticism scores tended to be higher in our female patients who needed specific medication for disease control vs. those who reached control without chronic medication. Furthermore, female patients with at least one pituitary insufficiency had lower values for agreeableness. Specific data allowing a direct comparison of the neuropsychological profile in female patients with acromegaly, in the light of influencing factors, are not available. However, female sex predicted a negative result for all scales except the appearance domain of the Acromegaly Quality of Life Questionnaire, according to Vandeva et al. (10).

An age- and sex- independent comparison between patients and controls revealed a less denying personality in patients with pituitary insufficiency. Among our hormonally controlled patients, neuroticism scores were higher in those having at least one pituitary insufficiency. Few previous investigations addressed the issue of concomitant pituitary deficiencies in acromegaly and all of these explored quality of life issues. Wexler et al. showed a significantly impaired quality of life in GH-deficient patients after treatment of acromegaly compared to GH-sufficient patients, as measured by the QoL-AGHDA, the Symptom Questionnaire Depression and SF36 questionnaires (19), while Vandeva et al. found that the absence of hypopituitarism independently predicted improvements in quality of life (10).

Disease activity had little impact on the observed neuropsychological changes in our study; only the score for the trait *nurturant* was higher in patients with active disease compared to controls, without further alterations in the psychological items of the NEO-FFI or the IIP-D inventory. There are few literature data analyzing the association of neuropsychological traits and disease activity. Ruchala et al. described some improvement of interpersonal relations under treatment with octreotide, with intensive repression of emotions (20). Current evidence suggests that a longterm cure or control of the disease activity does not necessarily improve the quality of life (21). Biermasz et al. was the first to report reduced general perceived well-being and quality of life in a cross-sectional study including 118 successfully treated acromegalic patients (8). A recent meta-analysis of published data revealed that disease-activity reflected by biochemical control measures yielded mixed, and therefore inconclusive results with respect to their effect on QoL (22). Sardella et al. suggested that successful treatment, resulting in complete remission, improved quality of life in acromegalic patients in the short term, at 6 months. However, the lack of correlation between the ACROQOL score after 24 months might suggest that factors like serum IGF-1 concentration play a role in determining the well-being of acromegalic patients (7).

It is not clear why psychological alterations are not limited to the duration of increased GH-exposure but persist in cured or controlled patients. Possible explanations for these findings may be related to alterations in the macroscopic brain architecture with increased global, left and right hippocampal gray matter and white matter volumes at the expense of cerebral spinal fluid (23, 24), as demonstrated in cerebral MRI. These persistent structural alterations could explain, at least in part, our neuropsychological findings, which were mainly independent of disease activity.

### Physical complaints, according to the GBB inventory

Comorbidities and pain are frequent in acromegaly. In our cohort, exhaustion and general complaints were more severe in patients than controls and were associated with pituitary insufficiency, a history of malignancy and coronary heart disease in the MV. Disease activity had no impact on exhaustion and general complaints, while joint complaints were not relevantly associated with any disease- or treatment-related variables or comorbidities. This suggests again that other factors, besides disease activity, and possibly long-term irreversible effects of GH-excess could be involved in the persistence of chronic complaints.

In line with this, in a retrospective, multicenter cohort study including 131 acromegalic patients within the German Pegvisomant Observational Study, perspiration, soft tissue swelling and perceived health improved after 1 year of pegvisomant therapy, while other symptoms such as headache, fatigue and joint pain remained largely unchanged over time, suggesting irreversible damage despite treatment (25).

Arthrosis occurred in 53.5% of patients, arterial hypertension in 36.4%, diabetes mellitus in 15.5% and coronary heart disease in 12.4%, while a history of malignancy was reported in 10% of patients. In the literature, among 118 patients in long-term remission after treatment, with a mean duration of remission of 12.0 ± 7.4 years, self-reported joint problems occurred in 77%, hypertension in 37%, diabetes mellitus in 11%, and a history of myocardial infarction in 9%. In accordance to our findings, the joint problems were not related to disease activity or age, previous or current disease-specific treatment. It has been already reported that joint complaints had a significant negative impact on the quality of life (9). Chronic pain appears to be a frequent problem in acromegaly. Investigating 81 acromegalic patients, a high prevalence of bodily pain (65%) and headache (65%), with an increased nociceptive pain component (80%) was found, without significant impact of modifiable factors like tumor size, genetic predisposition, previous surgery, irradiation or medical therapy, but with significant correlations with depression and quality of life (26).

A limitation of our study is the lack of a concurrent, real-life control group. However, we analyzed the psychologic and physical parameters based on manual data, with comparison to representative groups from the healthy general population. Due to the low prevalence of the disease, it was not possible to include more patients, despite the multi-center design. A further limitation is the cross-sectional, non-prospective design.

In conclusion, our multicenter study provides an extensive characterization of psychological traits, relationship patterns and subjective complaints in patients with acromegaly, with analysis of the impact of disease-modifying factors. The distinct neuropsychological profile and physical complaints are largely not dependent on hormonal control and seem to be in part disease specific, possibly due to long term GH excess, in part associated with chronic comorbidities. Concurrent pituitary insufficiency negatively impacts both the psychological profile and physical complaints.

Knowledge of the neuropsychological profile and subjective symptoms in view of the influencing factors could facilitate a more individual approach to patients with acromegaly and improve the care of disease-related aspects.

## Ethics statement

The work followed the ethical standards of the national research committee and the 1964 Helsinki declaration and its later amendments or comparable ethical standards. It was approved by the Ethics Committee of the Johannes Gutenberg University of Mainz, Germany. We obtained written informed consent from each patient, after full explanation of the purpose and nature of all procedures used and in line with the journal's policy.

## Author contributions

AZ collected and analyzed data and prepared the first draft of the paper. RZ and MB codesigned the inventory-based evaluation and critically revised the manuscript. MD, CS, CJS, UP, AZ, and JH collected data in other centers than Mainz and critically revised the manuscript. AD and BM collected, centralized and analyzed data in Mainz. GT was responsible for statistical analysis of the data. MW was responsible for conception and design and is the guarantor. All authors revised the paper critically for intellectual content and approved the final version. All authors agree to be accountable for the work and to ensure that any questions relating to the accuracy and integrity of the paper are investigated and properly resolved.

### Conflict of interest statement

The authors confirm independence from the sponsors. The content of the article has not been influenced by the sponsors. MW discloses that he received the research grant described under Funding, as a principal investigator. However, the authors declare that there is no conflict of interest that could be perceived as prejudicing the impartiality of the research reported. The remaining authors declare that the research was conducted in the absence of any commercial or financial relationships that could be construed as a potential conflict of interest.
