# Supplementary material for: Personality Traits and Physical Complaints in Patients With Acromegaly: A Cross Sectional Multi-Center Study With Analysis of Influencing Factors
Source: Front Endocrinol (Lausanne). 2018 Jul 17;9:391. doi: 10.3389/fendo.2018.00391 (PMC6056634; doi:10.3389/fendo.2018.00391)
Supplement: Supplementary file 1 [file Table_1.docx]

**Supplementary Table 1:** Predictive individual, clinical, treatment- and comorbidities-associated factors for the psychological traits of the NEO-FFI inventory

|  | **Multivariate analysis** | | |
| --- | --- | --- | --- |
| ***Neuroticism*** | **B** | **(95% CI)** | **p** |
| Age (years) | -0.02 | (-0.12 - 0.09) | 0.739 |
| Sex | -1.07 | (-3.66 - 1.52) | 0.413 |
| Disease activity | 1.6 | (-1.38 - 4.58) | 0.29 |
| Surgery | 0.46 | (-3.3 - 4.22) | 0.808 |
| Medication | 1.28 | (-1.36 - 3.92) | 0.34 |
| Radiation | 2.16 | (-1.95 - 6.28) | 0.3 |
| Pituitary insufficiency | 2.52 | (-0.26 - 5.3) | 0.075 |
| Coronary heart disease | 1.81 | (-2.25 - 5.86) | 0.379 |
| Arterial hypertension | 0.79 | (-2.12 - 3.69) | 0.593 |
| Diabetes | 0.32 | (-3.4 - 4.04) | 0.864 |
| History of malignancy | -2.3 | (-6.92 - 2.31) | 0.324 |
| Arthrosis | 1.13 | (-1.41 - 3.68) | 0.379 |
| ***Extraversion*** | **B** | **(95% CI)** | **p** |
| Age (years) | -0.04 | (-0.12 - 0.04) | 0.338 |
| Sex | -2.61 | (-4.63 - -0.6) | 0.112 |
| Disease activity | -2.09 | (-4.7 - 0.52) | 0.119 |
| Surgery | -1.5 | (-4.6 - 1.59) | 0.343 |
| Medication | 0.26 | (-1.91 - 2.42) | 0.817 |
| Radiation | -2.38 | (-6.17 - 1.42) | 0.222 |
| Pituitary insufficiency | -0.39 | (-2.23 - 1.46) | 0.683 |
| Coronary heart disease | -1.63 | (-4.7 - 1.44) | 0.301 |
| Arterial hypertension | 0.58 | (-1.88 - 3.05) | 0.645 |
| Diabetes | -0.76 | (-4.03 - 2.51) | 0.65 |
| History of malignancy | 2.24 | (-0.33 - 4.82) | 0.09 |
| Arthrosis | -0.56 | (-2.67 - 1.56) | 0.607 |
|  |  |  |  |
| ***Openness to experience*** | **B** | **(95% CI)** | **p** |
| Age (years) | 0.05 | (-0.05 - 0.14) | 0.336 |
| Sex | 0.14 | (-1.97 - 2.25) | 0.898 |
| Disease activity | -0.7 | (-3.39 - 1.99) | 0.612 |
| Surgery | -0.25 | (-3.23 - 2.73) | 0.87 |
| Medication | 0.44 | (-1.68 - 2.57) | 0.685 |
| Radiation | -1.24 | (-4.44 - 1.97) | 0.451 |
| Pituitary insufficiency | 0.29 | (-1.69 - 2.27) | 0.777 |
| Coronary heart disease | -2.47 | (-5.29 - 0.35) | 0.089 |
| Arterial hypertension | 0.24 | (-2.38 - 2.86) | 0.858 |
| Diabetes | 1.49 | (-1.52 - 4.51) | 0.333 |
| History of malignancy | -2.75 | (-5.44 - -0.06) | ***0.047*** |
| Arthrosis | -1.4 | (-3.38 - 0.58) | 0.169 |
| ***Agreeableness*** | **B** | **(95% CI)** | **p** |
| Age (years) | 0 | (-0.08 - 0.07) | 0.91 |
| Sex | -1.21 | (-2.97 - 0.54) | 0.178 |
| Disease activity | -0.29 | (-2.43 - 1.85) | 0.79 |
| Surgery | -0.09 | (-2.58 - 2.41) | 0.946 |
| Medication | 1.4 | (-0.34 - 3.13) | 0.117 |
| Radiation | -2.64 | (-5.66 - 0.38) | 0.089 |
| Pituitary insufficiency | -0.9 | (-2.71 - 0.91) | 0.332 |
| Coronary heart disease | -1.98 | (-4.64 - -0.32) | 0.13 |
| Arterial hypertension | -0.33 | (-2.33 - 1.66) | 0.742 |
| Diabetes | 1.09 | (-1.07 - 3.25) | 0.323 |
| History of malignancy | 3.21 | (0 - 6.42) | 0.053 |
| Arthrosis | -0.03 | (-1.78 - 1.73) | 0.977 |
| ***Conscientiousness*** | **B** | **(95% CI)** | **p** |
| Age (years) | 0.01 | (-0.08 - 0.09) | 0.878 |
| Sex | -1.68 | (-3.88 - 0.51) | 0.135 |
| Disease activity | -1.3 | (-3.76 - 1.16) | 0.303 |
| Surgery | -1.88 | (-4.97 - 1.21) | 0.235 |
| Medication | 0.87 | (-1.26 - 3) | 0.426 |
| Radiation | -2.5 | (-6.31 - 1.31) | 0.201 |
| Pituitary insufficiency | -1.92 | (-4.05 - 0.21) | 0.079 |
| Coronary heart disease | -1.32 | (-3.55 - 0.92) | 0.25 |
| Arterial hypertension | -0.98 | (-3.31 - 1.35) | 0.413 |
| Diabetes | -0.59 | (-3.1 - 1.91) | 0.643 |
| History of malignancy | 2.76 | (-0.04 - 5.55) | 0.056 |
| Arthrosis | -2.09 | (-3.23 - -0.16) | 0.067 |

B – increase or decrease of the dependent variable mean; 95% CI- 95% confidence interval
