# Supplementary material for: Personality Traits and Physical Complaints in Patients With Acromegaly: A Cross Sectional Multi-Center Study With Analysis of Influencing Factors
Source: Front Endocrinol (Lausanne). 2018 Jul 17;9:391. doi: 10.3389/fendo.2018.00391 (PMC6056634; doi:10.3389/fendo.2018.00391)
Supplement: Supplementary file 2 [file Table_2.docx]

**Supplementary Table 2:** Predictive individual, clinical, treatment- and comorbidities-associated factors for the psychological traits of the IIP-D inventory

|  | **Multivariate analysis** | | |
| --- | --- | --- | --- |
| ***Domineering*** | B | (95% CI) | p |
| Age (years) | 0 | (-0.01 - 0.01) | 0.726 |
| Sex | 0.06 | (-0.17 - 0.29) | 0.618 |
| Disease activity | 0.1 | (-0.17 - 0.37) | 0.472 |
| Surgery | 0.09 | (-0.25 - 0.43) | 0.609 |
| Medication | -0.08 | (-0.31 - 0.16) | 0.529 |
| Radiation | 0.3 | (-0.08 - 0.67) | 0.118 |
| Pituitary insufficiency | 0.1 | (-0.15 - 0.35) | 0.427 |
| Coronary heart disease | 0.24 | (-0.13 - 0.6) | 0.2 |
| Arterial hypertension | -0.09 | (-0.36 - 0.17) | 0.48 |
| Diabetes | -0.24 | (-0.58 - 0.09) | 0.152 |
| History of malignancy | -0.24 | (-0.65 - 0.18) | 0.265 |
| Arthrosis | 0 | (-0.23 - 0.23) | 0.994 |
| ***Vindictive/competing*** | B | (95% CI) | p |
| Age (years) | 0 | (-0.01 - 0.01) | 0.835 |
| Sex | 0.16 | (-0.03 - 0.36) | 0.103 |
| Disease activity | 0 | (-0.22 - 0.23) | 0.972 |
| Surgery | 0.06 | (-0.23 - 0.35) | 0.676 |
| Medication | 0.1 | (-0.1 - 0.3) | 0.313 |
| Radiation | 0.22 | (-0.09 - 0.53) | 0.167 |
| Pituitary insufficiency | 0.05 | (-0.16 - 0.26) | 0.643 |
| Coronary heart disease | 0.15 | (-0.16 - 0.46) | 0.351 |
| Arterial hypertension | -0.09 | (-0.31 - 0.13) | 0.413 |
| Diabetes | 0.12 | (-0.17 - 0.4) | 0.412 |
| History of malignancy | -0.21 | (-0.57 - 0.14) | 0.231 |
| Arthrosis | 0.2 | (0 - 0.39) | 0.078 |
| ***Cold/denying*** | B | (95% CI) | p |
| Age (years) | 0.01 | (0 - 0.02) | 0.202 |
| Sex | 0.21 | (-0.04 - 0.47) | 0.099 |
| Disease activity | 0.17 | (-0.12 - 0.46) | 0.257 |
| Surgery | 0.23 | (-0.14 - 0.6) | 0.225 |
| Medication | 0.17 | (-0.09 - 0.43) | 0.203 |
| Radiation | 0.1 | (-0.31 - 0.5) | 0.635 |
| Pituitary insufficiency | -0.15 | (-0.42 - 0.13) | 0.289 |
| Coronary heart disease | 0.09 | (-0.31 - 0.48) | 0.665 |
| Arterial hypertension | -0.1 | (-0.38 - 0.19) | 0.498 |
| Diabetes | -0.05 | (-0.42 - 0.31) | 0.771 |
| History of malignancy | -0.33 | (-0.78 - 0.13) | 0.156 |
| Arthrosis | 0.29 | (0.04 - 0.54) | 0.073 |
| ***Introverted/socially avoidant*** | B | (95% CI) | p |
| Age (years) | 0 | (-0.01 - 0.01) | 0.757 |
| Sex | 0.16 | (-0.1 - 0.42) | 0.228 |
| Disease activity | 0.22 | (-0.08 - 0.53) | 0.147 |
| Surgery | 0.16 | (-0.22 - 0.54) | 0.407 |
| Medication | 0.1 | (-0.17 - 0.36) | 0.48 |
| Radiation | 0.09 | (-0.33 - 0.5) | 0.688 |
| Pituitary insufficiency | -0.11 | (-0.39 - 0.17) | 0.436 |
| Coronary heart disease | 0.02 | (-0.4 - 0.43) | 0.936 |
| Arterial hypertension | -0.05 | (-0.35 - 0.24) | 0.718 |
| Diabetes | 0.03 | (-0.35 - 0.4) | 0.893 |
| History of malignancy | -0.28 | (-0.74 - 0.19) | 0.246 |
| Arthrosis | 0.3 | (0.04 - 0.56) | ***0.022*** |
| ***Non-assertive / insecure*** | B | (95% CI) | p |
| Age (years) | 0 | (-0.01 - 0.02) | 0.459 |
| Sex | -0.03 | (-0.3 - 0.23) | 0.797 |
| Disease activity | 0.23 | (-0.07 - 0.54) | 0.134 |
| Surgery | 0.15 | (-0.23 - 0.54) | 0.439 |
| Medication | 0.19 | (-0.08 - 0.47) | 0.161 |
| Radiation | 0.01 | (-0.42 - 0.43) | 0.975 |
| Pituitary insufficiency | 0.03 | (-0.25 - 0.32) | 0.824 |
| Coronary heart disease | -0.26 | (-0.68 - 0.15) | 0.212 |
| Arterial hypertension | -0.01 | (-0.31 - 0.29) | 0.934 |
| Diabetes | -0.07 | (-0.45 - 0.32) | 0.73 |
| History of malignancy | -0.09 | (-0.57 - 0.38) | 0.694 |
| Arthrosis | 0.19 | (-0.08 - 0.45) | 0.163 |
| ***Exploitable/permissive*** | B | (95% CI) | p |
| Age (years) | 0 | (-0.01 - 0.01) | 0.564 |
| Sex | -0.1 | (-0.33 - 0.13) | 0.38 |
| Disease activity | 0.14 | (-0.12 - 0.4) | 0.279 |
| Surgery | 0.11 | (-0.22 - 0.43) | 0.526 |
| Medication | 0.17 | (-0.06 - 0.4) | 0.14 |
| Radiation | -0.13 | (-0.49 - 0.23) | 0.467 |
| Pituitary insufficiency | 0.06 | (-0.19 - 0.3) | 0.652 |
| Coronary heart disease | -0.11 | (-0.46 - 0.24) | 0.543 |
| Arterial hypertension | 0.05 | (-0.2 - 0.3) | 0.702 |
| Diabetes | 0.04 | (-0.29 - 0.36) | 0.816 |
| History of malignancy | -0.04 | (-0.44 - 0.37) | 0.854 |
| Arthrosis | 0.02 | (-0.2 - 0.24) | 0.847 |
| ***Nurturant*** | B | (95% CI) | p |
| Age (years) | 0.01 | (0 - 0.01) | 0.191 |
| Sex | -0.1 | (-0.31 - 0.11) | 0.343 |
| Disease activity | 0.1 | (-0.14 - 0.34) | 0.415 |
| Surgery | 0.09 | (-0.21 - 0.39) | 0.564 |
| Medication | 0.17 | (-0.04 - 0.38) | 0.112 |
| Radiation | -0.33 | (-0.66 - 0) | 0.062 |
| Pituitary insufficiency | -0.16 | (-0.39 - 0.06) | 0.145 |
| Coronary heart disease | -0.09 | (-0.42 - 0.23) | 0.577 |
| Arterial hypertension | -0.01 | (-0.24 - 0.22) | 0.927 |
| Diabetes | -0.1 | (-0.4 - 0.19) | 0.486 |
| History of malignancy | 0.09 | (-0.28 - 0.46) | 0.623 |
| Arthrosis | 0.04 | (-0.17 - 0.24) | 0.716 |
| ***Intrusive*** | B | (95% CI) | p |
| Age (years) | 0 | (-0.01 - 0.01) | 0.855 |
| Sex | -0.04 | (-0.28 - 0.2) | 0.754 |
| Disease activity | 0.07 | (-0.2 - 0.35) | 0.609 |
| Surgery | 0.07 | (-0.28 - 0.42) | 0.698 |
| Medication | 0.04 | (-0.21 - 0.28) | 0.774 |
| Radiation | 0.03 | (-0.36 - 0.41) | 0.896 |
| Pituitary insufficiency | 0.05 | (-0.2 - 0.31) | 0.677 |
| Coronary heart disease | 0.04 | (-0.34 - 0.41) | 0.851 |
| Arterial hypertension | 0.06 | (-0.21 - 0.33) | 0.659 |
| Diabetes | -0.13 | (-0.48 - 0.21) | 0.443 |
| History of malignancy | -0.05 | (-0.48 - 0.37) | 0.803 |
| Arthrosis | 0.02 | (-0.21 - 0.26) | 0.838 |
| ***IIP D total*** | B | (95% CI) | p |
| Age (years) | 0 | (0 - 0.01) | 0.382 |
| Sex | 0.04 | (-0.12 - 0.2) | 0.639 |
| Disease activity | 0.13 | (-0.06 - 0.32) | 0.172 |
| Surgery | 0.1 | (-0.13 - 0.34) | 0.385 |
| Medication | 0.11 | (-0.05 - 0.28) | 0.172 |
| Radiation | 0.05 | (-0.21 - 0.3) | 0.715 |
| Pituitary insufficiency | -0.01 | (-0.18 - 0.17) | 0.929 |
| Coronary heart disease | -0.01 | (-0.26 - 0.25) | 0.962 |
| Arterial hypertension | -0.03 | (-0.21 - 0.16) | 0.779 |
| Diabetes | -0.06 | (-0.29 - 0.17) | 0.626 |
| History of malignancy | -0.12 | (-0.41 - 0.17) | 0.411 |
| Arthrosis | 0.12 | (-0.03 - 0.28) | 0.123 |

###

B – increase or decrease of the dependent variable mean; 95% CI- 95% confidence interval
