# Supplementary material for: Personality Traits and Physical Complaints in Patients With Acromegaly: A Cross Sectional Multi-Center Study With Analysis of Influencing Factors
Source: Front Endocrinol (Lausanne). 2018 Jul 17;9:391. doi: 10.3389/fendo.2018.00391 (PMC6056634; doi:10.3389/fendo.2018.00391)
Supplement: Supplementary file 3 [file Table_3.docx]

**Supplementary Table 3:** Predictive individual, clinical, treatment and comorbidities-associated factors for the physical complaints according to the GBB inventory

| ***Exhaustion*** | B | (95% CI) | p |
| --- | --- | --- | --- |
| Age (years) | -0.09 | (-0.16 - -0.02) | 0.117 |
| Sex | -0.47 | (-2.59 - 1.66) | 0.665 |
| Disease activity | 0.63 | (-1.73 - 2.98) | 0.598 |
| Surgery | 1.74 | (-1.56 - 5.03) | 0.299 |
| Medication | 1.05 | (-1.2 - 3.3) | 0.355 |
| Radiation | -0.03 | (-2.99 - 2.93) | 0.983 |
| Pituitary insufficiency | 2.76 | (0.54 - 4.98) | ***0.015*** |
| Coronary heart disease | 4.45 | (1.11 - 7.79) | ***0.009*** |
| Arterial hypertension | 0.85 | (-1.31 - 3) | 0.438 |
| Diabetes | 1.81 | (-1.01 - 4.63) | 0.206 |
| History of malignancy | 0.91 | (-2.64 - 4.45) | 0.614 |
| Arthrosis | 1.2 | (-0.87 - 3.28) | 0.253 |
| ***Gastric complaints*** | B | (95% CI) | p |
| Age (years) | 0 | (-0.05 - 0.05) | 0.988 |
| Sex | -0.6 | (-1.9 - 0.71) | 0.374 |
| Disease activity | 0.51 | (-1.1 - 2.12) | 0.535 |
| Surgery | 0.43 | (-1.45 - 2.31) | 0.655 |
| Medication | -0.05 | (-1.36 - 1.26) | 0.942 |
| Radiation | 0.65 | (-1.4 - 2.7) | 0.537 |
| Pituitary insufficiency | 1.5 | (-0.1 - 3.11) | 0.069 |
| Coronary heart disease | -0.41 | (-2.35 - 1.54) | 0.681 |
| Arterial hypertension | 0.32 | (-1.15 - 1.79) | 0.674 |
| Diabetes | 0.75 | (-1 - 2.5) | 0.404 |
| History of malignancy | 3.03 | (1 - 5.07) | 0.156 |
| Arthrosis | -0.63 | (-2.03 - 0.76) | 0.376 |
| ***Joint complaints*** | B | (95% CI) | p |
| Age (years) | 0 | (-0.07 - 0.07) | 0.981 |
| Sex | -1.37 | (-3.41 - 0.68) | 0.187 |
| Disease activity | 0.23 | (-2.03 - 2.5) | 0.838 |
| Surgery | 2.53 | (-0.64 - 5.7) | 0.117 |
| Medication | 0 | (-2.17 - 2.16) | 0.997 |
| Radiation | 0.5 | (-2.34 - 3.34) | 0.728 |
| Pituitary insufficiency | 1.53 | (-0.6 - 3.66) | 0.158 |
| Coronary heart disease | 2.47 | (-0.74 - 5.68) | 0.13 |
| Arterial hypertension | 0.81 | (-1.26 - 2.88) | 0.44 |
| Diabetes | 2.24 | (-0.47 - 4.95) | 0.105 |
| History of malignancy | 2.79 | (-0.62 - 6.2) | 0.108 |
| Arthrosis | 0.85 | (-1.14 - 2.85) | 0.4 |
|  |  |  |  |
| ***Heart complaints*** | B | (95% CI) | p |
| Age (years) | 0 | (-0.05 - 0.05) | 0.965 |
| Sex | -1.1 | (-2.48 - 0.28) | 0.116 |
| Disease activity | 0.97 | (-0.58 - 2.53) | 0.217 |
| Surgery | -0.39 | (-2.54 - 1.77) | 0.724 |
| Medication | 0.52 | (-0.95 - 2) | 0.485 |
| Radiation | 0.03 | (-1.89 - 1.95) | 0.976 |
| Pituitary insufficiency | 0.45 | (-1.01 - 1.92) | 0.541 |
| Coronary heart disease | 1.8 | (-0.37 - 3.97) | 0.103 |
| Arterial hypertension | 0.85 | (-0.56 - 2.26) | 0.236 |
| Diabetes | 0.75 | (-1.08 - 2.58) | 0.418 |
| History of malignancy | 3.23 | (0.93 - 5.54) | ***0.006*** |
| Arthrosis | 0.12 | (-1.23 - 1.47) | 0.857 |
| ***GBB-total*** | B | (95% CI) | p |
| Age (years) | -0.1 | (-0.29 - 0.1) | 0.336 |
| Sex | -3.39 | (-8.78 - 1.99) | 0.22 |
| Disease activity | 2.26 | (-3.9 - 8.42) | 0.473 |
| Surgery | 4.23 | (-3.22 - 11.68) | 0.268 |
| Medication | 1.35 | (-4.52 - 7.22) | 0.653 |
| Radiation | 1.36 | (-7.02 - 9.74) | 0.75 |
| Pituitary insufficiency | 6.26 | (0.28 - 12.23) | ***0.043*** |
| Coronary heart disease | 8.45 | (-1.21 - 18.12) | 0.089 |
| Arterial hypertension | 2.52 | (-3.23 - 8.26) | 0.392 |
| Diabetes | 5.74 | (-1.79 - 13.28) | 0.138 |
| History of malignancy | 10.07 | (0.87 - 19.27) | ***0.034*** |
| Arthrosis | 1.54 | (-4.22 - 7.29) | 0.602 |

B – increase or decrease of the dependent variable mean; 95% CI- 95% confidence interval

### 
